# *Glutathione S-transferase Omega 2* Genetic Polymorphism and Risk of Hepatic Failure that Lead to Liver Transplantation in Iranian Population

**Published:** 2013-02-01

**Authors:** M. Khosravi, I. Saadat, M. H. Karimi, B. Geramizadeh, M. Saadat

**Affiliations:** 1 *Department of Biology, College of Sciences, Shiraz University, Shiraz 71454, Iran*; 2 *Institute of Biotechnology, Shiraz University, Shiraz, Iran*; 3 *Transplant Research Center, Shiraz University of Medical Sciences, Shiraz, Iran*

**Keywords:** Hepatic failure, Liver transplantation, Glutathione S-transferase

## Abstract

Background: Liver transplantation is the treatment of choice for both acute and chronic hepatic failure. GSTs family is one of the most important genes in phase II detoxification interfering with the xenobiotics and free radical metabolism. *GSTO2* (N142D) is a member of this family the polymorphism of which may influence the metabolism of active components and free radicals and may contribute to hepatic failure.

Objective: To investigate the association between *GSTO*2 genetic polymorphism and the susceptibility of hepatic failure that would lead to liver transplantation.

Methods: This case-control study included 330 healthy people and 302 patients with liver transplantation as a result of hepatic failure. To determine the variants of *GSTO2, *we used polymerase chain reaction-restriction fragment length polymorphism (PCR-RFLP) method.

Results: There was a significant association between D allele and hepatic failure, thus, people with DD genotype are more susceptible to develop heaptic failure leading to liver transplantation (OR=1.8, 95% CI: 1.10–2.95, p=0.02). We also observed that male sex increases the chance of hepatic failure (OR=2.69, 95% CI: 1.95–3.71, p=0.001).

Conclusion: D allele may reduce the detoxification ability of liver so people with mutant D allele are more prone to develop hepatic failure.

## INTRODUCTION

Liver transplantation is the treatment of choice for those with acute and chronic hepatic failure. Hepatic failure is characterized by reduced liver function, muscle loss, fatigue, encephalopathy, signs of portal hypertension, poor blood clotting and jaundice. One of the important functions of liver is its detoxification ability [1]. Cytochorome P450 plays an important role in phase I detoxification. It is the first enzymatic defense against xenobiotics using O_2_ and NADH for adding active group to produce polar components that may be more toxic than the first component [1, 2]. If these active components, free radicals and reactive oxygen species (ROS) which are produced in our body, are not neutralized rapidly by phase II detoxification, they may damage the hepatocyte through damaging DNA, RNA and protein [2], leading to liver disease and the patient may need liver transplantation. In phase II detoxification, hepatocytes can add some groups such as glutathione to the noxious molecules to increase their water solubility to be excreted in urine and bile [1]. Glutathione is a substance that neutralizes free radicals and active components through glutathione S-transferases (GSTs). A reduction in the glutathione level or GST enzyme activity leads to hepatocyte damage. Glutathione S-transferase family is the main enzyme in phase II detoxification and plays a critical role in xenobiotic and drug metabolism through glutathione binding catalysis. Polymorphism of GST family can affect the detoxification capacity and thus may cause cancer or other diseases [3]. 

The GSTs were categorized to several classes including omega (GSTO), zetta (GSTZ), mu (GSTM) and theta (GSTT) classes. GSTO class has two members—GSTO1 and GSTO2. The human GSTO2 is a protein with 243 amino acid residues with a Cysteine in its active site; it plays a major role in detoxification of arsenic. In human, the *GSTO2* is polymorphic with an N142D substitution in the coding region [4]. It is reported that the *GSTO2* Asp142 (D142) variant allozyme showed 20% reduction in level of expression compared with the level of the *GSTO2* wild type (N142) allozyme [5].

*GSTO2* is expressed by whole cell body especially liver, kidney, skeletal muscles and prostate [4]. Polymorphism of *GSTO2* (N142D) is associated with many cancers such as colorectal [6], gastric [7] and ovarian cancers [8]. This polymorphism would also have a role in asthma [9, 10] and hepatocellular carcinoma [11].

The association of *GSTO2* (N142D) and hepatic failure has not been examined so far. Therefore, we conducted this study to investigate the association between *GSTO2 *genetic polymorphism and the susceptibility of hepatic failure that would lead to liver transplantation.

## MATERIALS AND METHODS

Buffy coat samples from 302 liver transplanted patients were collected from Namazi Hospital, Shiraz, southern Iran between 2007 and 2011. Blood samples were also taken from a control group consisting of 330 healthy age-matched people with no cancer or liver problem from Shiraz Transfusion Center. Medical information was collected from patients’ medical report. The study was approved by the Ethics Committee of Shiraz University. Informed consent was obtained from all participants.

**Figure 1:**
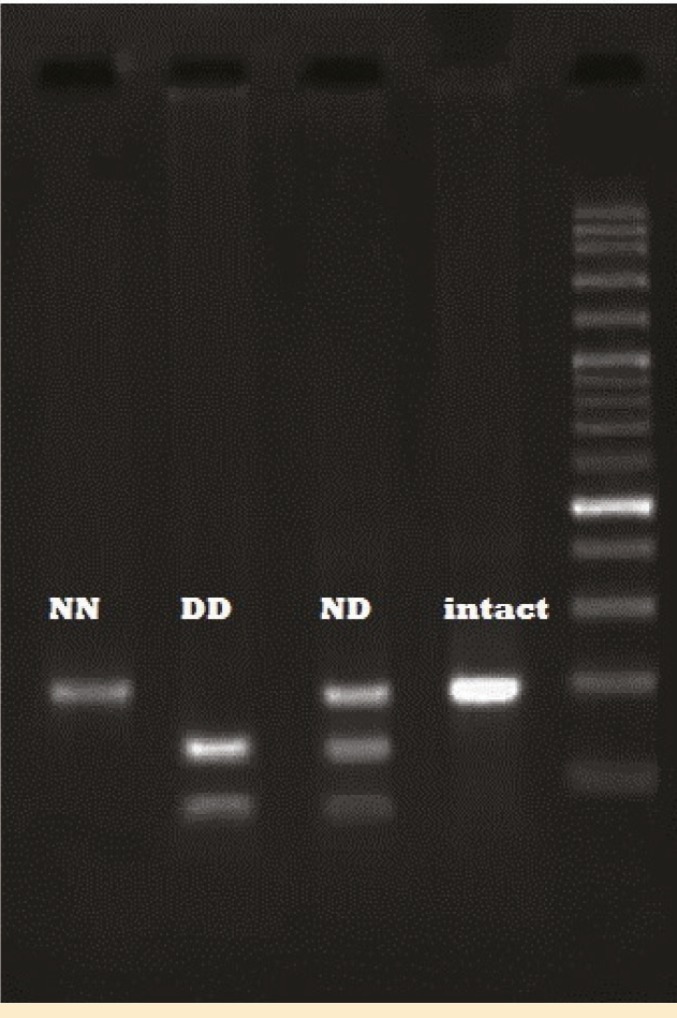
Genotyping of the *GSTO2* (N142D) polymorphism by *MboI* RFLP. From right to left the lanes are DNA size marker (100 bp ladder), intact (185 bp), ND genotype (185, 122 and 63 bp), DD genotype (122, 63 bp) and NN genotype (185 bp), respectively.

DNA was extracted with DNP kit (CinnaGen Inc) according to manufacturer’s instruction. The genotype of *GSTO2* N142D was determined by PCR-RFLP method as described previously [7]. The PCR-RFLP products corresponding to NN, ND and DD genotypes were separated by electrophoresis on 2% agarose gel (Fig 1).

To test the consistency of the genotyping and to detect any possible misgenotyping, 10% of samples selected at random were retested.

Statistical analysis 

SPSS17.0 (SPSS Inc., Chicago, IL, USA) was used for data analyses. We used *Studet’s t* test for comparing means of two continuous variables. χ^2^ test was used to study the deviation from Hardy-Weinberg equilibrium between the observed and expected genotype frequencies in healthy population. We used binary logistic regression analysis to determine the independent risk factors of developing liver problems. A p value <0.05 was considered statistically significant.

## RESULTS

**Figure 2:**
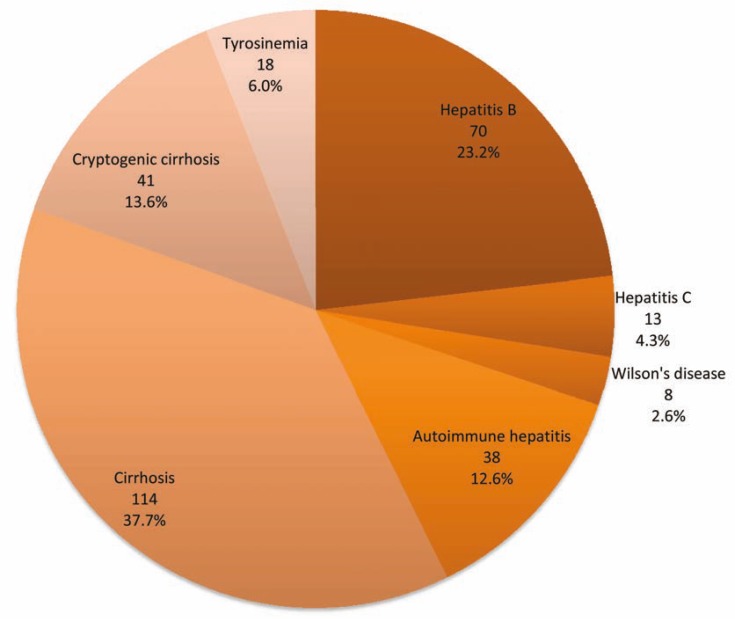
The underlying causes of hepatic failure

There was no discrepancy in retesting of results. The mean±SD age of the case group was 30.3±18.7 years. The mean±SD age of the control group was 36.3±12.7 years. Most of patients (37.7%) had cirrhosis; the rest had other underlying diseases (Fig 2).

The allelic frequency of 142D, and 142N in our control group was estimated to be 0.347, and 0.653, respectively. The genotypes of *GSTO2* N142D in the control group were in Hardy-Weinberg equilibrium (χ2=0.0042, df=1, p>0.05). Statistical analyses showed a significant association between *GSTO2 *(N142D) polymorphism and hepatic failure that led to LT (Table 1). The *GSTO2 *ND (OR=1.61, 95% CI: 1.14–2.27, p=0.007) and DD (OR=1.8, 95% CI: 1.10–2.95, p=0.02) genotypes were associated with an increased risk of developing hepatic failure that led to LT as compared to NN genotype. Furthermore, we found that male sex was more prone to develop hepatic failure that led to LT (OR=2.69, 95% CI: 1.95–3.71, p=0.001).

**Table 1:** Association of *GSTO2 *(N142D) and hepatic failure that led to LT

**Genotype**	**LT (%)**	**Healthy (%)**	**OR (95% CI)**
**NN**	94 (31.1)	141 (42.7)	Ref (1)
**ND**	160 (53.0)	149 (45.2)	11.611(1.14–2.27)
**DD**	48 (15.9)	40 (12.1)	1.8(1.10–2.95)

## DISCUSSION

Several studies have shown that the polymorphism of *GSTO2* has a role in the colorectal, gastric and hepatocellular carcinoma [6-7, 11]. In 2011, it was shown that there is no association between *GSTM1* and *GSTT1* polymorphism and acute rejection of liver transplant [12]. However, the association of *GSTO2 *polymorphism and hepatic failure that would lead to LT has not yet been examined. In this study we showed that augmentation of D allele can increase the risk of hepatic failure that led to LT. It is reported that the GSTO2 Asp142 (D142) variant allozyme expresses 20% less than that of the GSTO2 wild type (N142) allozyme [5]. It is possible that the wild type genotype (NN) can metabolize the active components and free radical better, more rapidly and efficiently than the mutant genotypes (ND, DD), so that these noxious components have little time to damage hepatocytes; thus, a person with the mutant genotype is more sensitive to these components—peoples with ND and DD genotype for *GSTO2* are more prone to develop hepatic failure^.^

In our study, we observed that male sex was more prone to develop hepatic failure that led to LT. Previous studies showed that ovariectomy in rat would enhance formation of ROS which is blocked by progesterone [13, 14]. Since the progesterone level is higher in women than men, it is reasonable to presume that men have higher levels of ROS and thus are more prone to develop hepatic failure. However, since this is multifactorial, it is hard to conclude prematurely and further studies are needed.
